# Inhibition of IKKβ by celastrol and its analogues – an *in silico* and *in vitro* approach

**DOI:** 10.1080/13880209.2016.1241809

**Published:** 2016-12-08

**Authors:** Karpagam Veerappan, Sathishkumar Natarajan, Purushoth Ethiraj, Umashankar Vetrivel, Shila Samuel

**Affiliations:** a Department of Biochemistry, VRR Institute of Biomedical Science (Affiliated to University of Madras), Chennai, Tamilnadu, India;; b Department of Horticulture, Sunchon National University, Suncheon, Republic of Korea;; c Department of Medical Research, SRM Medical College Hospital and Research Centre, SRM University, Kattankulathur, Tamilnadu, India;; d Center for Bioinformatics, Vision Research Foundation, Sankara Nethralaya, Chennai, Tamilnadu, India

**Keywords:** Alzheimer’s disease, neuroinflammation, amyloid-β, molecular docking, ADMET

## Abstract

**Context:** Alzheimer’s disease (AD) is the most common form of dementia affecting the aged population and neuroinflammation is one of the most observed AD pathologies. NF-κB is the central regulator of inflammation and inhibitor κB kinase (IKK) is the converging point in NF-κB activation. Celastrol is a natural triterpene used as a treatment for inflammatory conditions.

**Objective:** This study determines the neuroprotective and inhibitory effect of celastrol on amyloid beta_1-42_ (Aβ_1-42_) induced cytotoxicity and IKKβ activity, respectively.

**Materials and methods:** Retinoic acid differentiated IMR-32 cells were treated with celastrol (1 μM) before treatment with Aβ_1-42_ (IC_30_ 10 μM) for 24 h. The cytotoxicity and IKK phosphorylation were measured by MTT and western blotting analysis, respectively. We screened 36 celastrol analogues for the IKKβ inhibition by molecular docking and evaluated their drug like properties to delineate the neuroprotective effects.

**Results:** Celastrol (1 μM) inhibited Aβ_1-42_ (10 μM) induced IκBα phosphorylation and protected IMR-32 cells from cell death. Celastrol and 25 analogues showed strong binding affinity with IKKβ as evidenced by strong hydrogen-bonding interactions with critical active site residues. All the 25 analogues displayed strong anti-inflammatory properties but only 11 analogues showed drug-likeness. Collectively, molecule 15 has highest binding affinity, CNS activity and more drug likeness than parent compound celastrol.

**Discussion and conclusion:** The decreased expression of pIκBα in celastrol pretreated cells affirms the functional representation of inhibited IKKβ activity in these cells. The neuroprotective potentials of celastrol and its analogues may be related to IKK inhibition.

## Introduction

Alzheimer’s disease (AD) is the most common form of dementia affecting the elderly population. The extracellular accumulation of amyloid beta (Aβ) peptide deposits (neuritic plaques) and intracellular neurofibrillary tangles (NFT) are the characteristic microscopic lesions observed in AD brains (Hardy & Selkoe 2002). Although Aβ (both plaques and oligomers) formation has long been considered as the upstream trigger or causative agent of AD (Serrano-Pozo et al. 2011), therapies targeting Aβ pathways have failed early in the clinical trials (Giacobini & Gold 2013; Castello et al. 2014). Thus, there is an urgent demand for the prevention or disease modifying therapies for AD.

Chronic neuroinflammation is another characteristic pathology of AD as evident by the presence of activated microglia and astrocytes around the neuritic plaques in addition to increased pro-inflammatory signalling (Vehmas et al. 2003; Glass et al. 2010; Zotova et al. 2010). Therefore, decreasing neuroinflammation could be an alternative strategy in AD drug discovery. NF-κB is a crucial transcriptional regulator of inflammatory response including brain. Accumulating evidences reveal that NF-κB signalling in neurons, microglia and astrocytes forms a vicious cascade of inflammatory events in mediating neuronal loss in AD (Mattson & Camandola 2001).

The inhibitor κB kinase (IKK) complex is the key enzyme in the activation of NF-κB. The IKK complex consists of three subunits: two catalytic subunits (IKKα and IKKβ) and a regulatory subunit IKKγ also termed as NEMO (Gilmore 2006; Perkins 2007). These proteins predominantly exist as IKKα/IKKβ heterodimer associated with IKKγ. In the resting state, the NF-κB is trapped in the cytoplasm by an inhibitor protein IκB. Upon stimulation, IκB is site specifically phosphorylated by the IKK and targeted for proteasome degradation, then, NF-κB translocates into the nucleus and activates target genes. Although both IKKα and IKKβ are able to phosphorylate IκB, IKKβ has been shown to be predominant in canonical pathway activating NF-κβ in response to Aβ (Barger et al. 1995; Barger & Mattson 1996). Consequently, IKKβ represents as an attractive target in the NF-κB pathway for the development of anti-inflammatory-based AD therapeutics.

Celastrol, derived from the root of “Thunder god vine” (*Tripterygenium wilfordii*), is a potent inhibitor of IKK-NF-κB signalling. Recent evidence has also shown celastrol as a promising therapeutics in models of AD, amyotrophic lateral sclerosis, Parkinson's disease and Huntington’s disease (Cleren et al. 2005; Kiaei et al. 2005; Paris et al. 2010). Although celastrol has potential therapeutic value, its toxicity is a major problem in drug discovery. To overcome this, structural modifications of celastrol were produced in recent years (Sun et al. 2010; Tang et al. 2014, 2015).

We studied the molecular interaction between the celastrol and its 36 analogues with IKKβ and the effect of celastrol on Aβ induced IKKβ inhibition in differentiated IMR-32 cells. The drug like properties of the celastrol and its analogues were also evaluated. 25 celastrol analogues were found to inhibit IKKβ and 11 of them demonstrated drug-like properties.

## Materials and methods

### Cell culture and differentiation

IMR-32 cells were obtained from NCCS (Pune, India) and grown in DMEM (Sigma Aldrich, St. Louis, MO) supplemented with 10% Fetal Bovine Serum (FBS) and 1% antibiotics (Penicillin 50 IU/mL, Streptomycin 3.5 μg/mL) (GIBCO, Grand Island, NY). IMR-32 cells were plated on 10 mm cell culture dishes coated with laminin (SPL Life Sciences Co., Ltd. Korea). After 24 h of seeding, cells were differentiated with retinoic acid (RA, Sigma Aldrich, St. Louis, MO) to the final concentration of 10 μM for 7 days.

### Cell proliferation assay

Differentiated IMR-32 cells were seeded in 96-well plates at a density of 1 × 10^4^ cells/well in 200 μL of DMEM containing 10% FBS and incubated overnight. To study the effect of celastrol on Aβ_1-42_ induced cell damage, cells were pre-incubated with celastrol (1 μM) (Cayman Chemical Company, USA, purity ≥98%) for 12 h and then Aβ_1-42_ (10 μM) (Sigma Aldrich, St. Louis, MO) was added to the medium for additional 24 h. A control (untreated) containing serum-free medium was also evaluated. Then plates were incubated with MTT solutions (0.5 mg/mL) for 3 h at 37 °C. The formazan was dissolved in 150 μL/well dimethyl sulfoxide (DMSO) and the absorbance was detected at 550 nm using microplate reader (Bio Rad, CA, USA). Cell viability was expressed as a percentage of untreated cells.

### Western blotting

The cells were seeded in 10 mm Petri plates in 1.5 mL of DMEM containing 10% FBS overnight. After treatment with celastrol and Aβ_1-42,_ cells were lysed by the addition of cold RIPA buffer (150 mM NaCl, 50 mM Tris HCl, 0.1% SDS, 1% Triton X-100, 1 mM PMSF, 2 mM NaF, Na_3_VO_4_, β-glycerophosphate and 2 mM EDTA) and fresh protease inhibitor cocktail, and cell lysate was centrifuged at 14,000 rpm at 4 °C for 20 min. The supernatant was harvested and analyzed for protein content using BCA method (Pierce, IL, USA). Protein was denatured in sample buffer, then separated on 12% SDS-PAGE, and transferred to polyvinylidene difluoride (PVDF) membranes. The blots were blocked for 2 h at room temperature with Tris-buffered saline (TBS, 50 mM Tris-HCl, pH 7.5, 150 mM NaCl) containing 5% non-fat milk. The blots were washed three times with TBST (50 mM Tris-HCl, pH 7.5, 150 mM NaCl, and 0.02% Tween 20) and incubated with p-IκBα and p-p65 primary antibodies (Cell Signalling Tech Inc., USA) (1:1000 dilutions) at 4 °C overnight. The blots were incubated for 1 h at room temperature with secondary antibodies (1:5000 dilutions), and detected by ECL detection reagent. To ensure that equal amounts of sample protein were applied for electrophoresis, GAPDH was used as an internal control. Densitometry analysis was done using Image lab™ (Bio-Rad, CA, USA) software.

### Celastrol compounds collection and preparation

**Figure 1.**
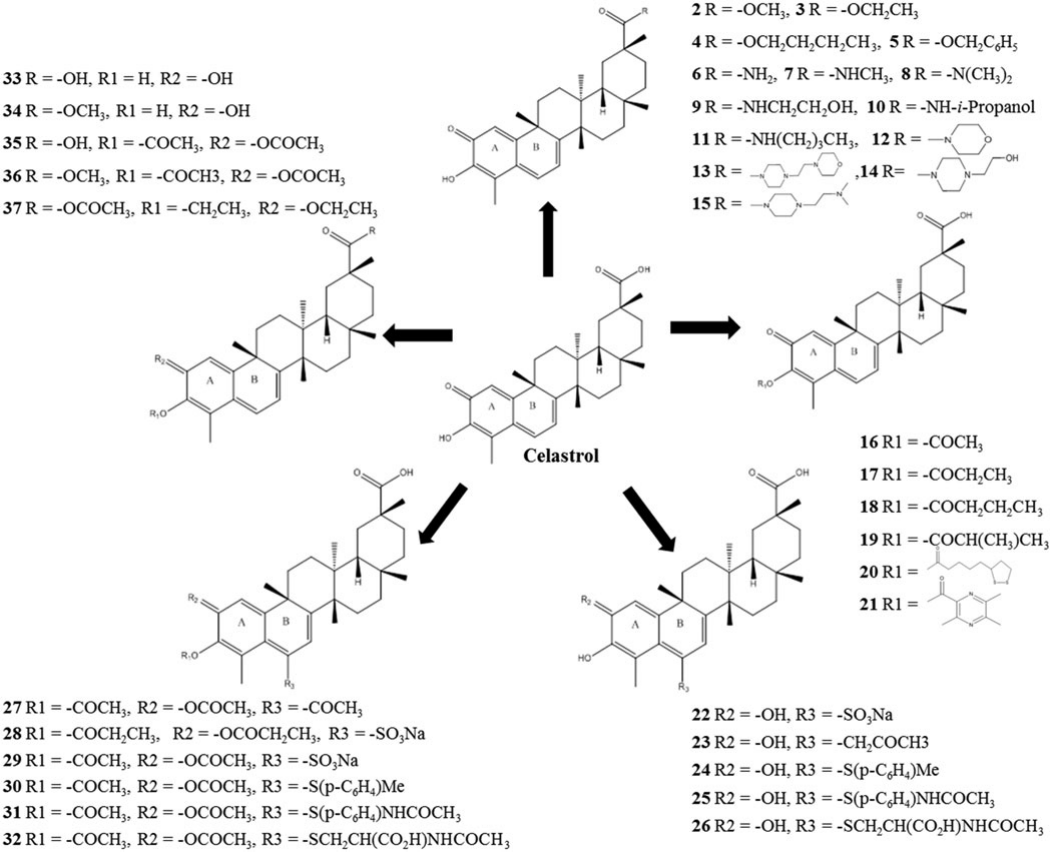
Chemical structure of celastrol and its analogues.

The structure of celastrol and its 36 celastrol analogues (Figure 1) were collected from the literature (Sun et al. 2010; Tang et al. 2014, 2015). The collected celastrol compounds were drawn in two dimensional (2D) structure using ChemSketch program (http://www.acdlabs.com, Advanced Chemistry Development, Inc. Toronto, Ontario, Canada). These 2D structures were converted into three dimensional (3D) structures format by importing into Accelrys Discovery Studio 3.5 visualizer (DS 3.5) (DS, http://www.accelrys.com; Accelrys, Inc. San Diego, CA, USA) and saved in .mol format for each compound. In addition, these molecules were optimized using the Conjugate Gradients method (Barzilai & Borwein 1988) followed by Steepest Descent (Sterck 2013) in 200 steps using PyRx program (Wolf 2009). The minimization step was carried out using Universal force field (UFF) (Rappe et al. 1992). These prepared molecules were used for further analysis.

### Protein preparation and molecular interaction studies

The 3D structure of IKKβ protein was retrieved from Protein Data Bank (PDB ID: 4KIK). This protein structure was determined by X-ray crystallography with 2.83 Å resolution (Liu et al. 2013). For docking studies, the K-252A (KSA) inhibitor, co-crystallized in the PDB structure was extracted from IKKβ B chain and was used as a reference ligand. The binding orientation between 4KIK and KSA was considered to be the favourable binding site. The KSA molecule was optimized similarly as celastrol analogues using the Conjugate Gradients, PyRx program and UFF. The water molecules were removed and hydrogen atoms were added to the protein.

### Molecular interaction study

Molecular docking is an efficient tool for screening and identification of binding affinity of ligand to protein active site residues. In this study, docking simulation was performed using the advanced automated docking program AutoDock Vina (Trott & Olson 2010). This is a new open source program which can be used for drug discovery, molecular docking, and virtual screening, offering multi-core capability, high performance, enhanced accuracy, and ease of use. The binding affinity was predicted by implementing modified and expanded versions of ChemScore18 scoring functions. The prepared celastrol analogues were docked with flexible IKKβ active sites residues (Liu et al. 2013) for generating suitable binding poses for each compound. The potential binding interaction was identified based on binding affinity scores and hydrogen bond interactions between IKKβ and each celastrol analogues. The results of each molecule were analyzed by AutoDock tools graphical interface. The corresponding docked complex files (protein-ligand) were saved carefully for each molecule and used for further validation.

### Docking validation and pharmacophore feature extraction

Initially, the co-crystallized known inhibitor KSA was extracted and re-docked with protein active site to determine the reproducibility. The binding affinity scores were re-evaluated using the X-score version 1.3 program (MI, USA) (Wang et al. 2002) for each docking complexes. Along with binding affinity, other interactions such as hydrophobic pair score, hydrophobic match score, hydrophobic surface score, predicted mean binding affinity from the docking complexes of each molecule were calculated. Furthermore, the pharmacophore features present in each celastrol-IKKβ complexes were identified using the LigandScout program version 3.12 (Trial version, Vienna, Austria) (Wolber & Langer 2005).

### Pharmacokinetic properties prediction

The 2D structures of celastrol and the analogues were used for predicting pharmacokinetic properties such as absorption, distribution, metabolism, excretion, and toxicity (ADMET). The QikProp module encoded in Schrodinger program (http://www.schrodinger.com) was used to identify pharmacologically active molecules from celastrol dataset. Also, range of acquired drug-like properties of these compounds was compared with those 95% of the known drugs. The physiochemical and pharmacokinetic properties predicted in this study included serum protein binding (QPlogKhsa), logP for octanol/water (QPlogpo/w), blood brain barrier level (QPlogBB), and human oral absorption in gastrointestinal tract (QP %) (Harvey 2008). Further, drug likeness properties were calculated using Lipinski’s rule of five (Lipinski 2004).

### In-silico biological activity prediction

A computational program called Prediction of Activity Spectra for Substances (PASS) was used for predicting possible biological activity (Poroikov et al. 2003; Siraj et al. 2015) from the chemical descriptor of each molecule. For this study, all celastrol compounds in .mol format were used as input structure. This method produced list of biological activity along with the values of probability of active (Pa) and probability of inactive (Pi). The accuracy of biological activity was carefully measured based on Pa value. Pa <0.3 was considered as the molecule having less chance of probability to act like biological function.

### Data analysis

The values are expressed as means ± standard deviation (SD). Differences between the groups were assessed by Student’s *t*-test. *p* values of <0.05 was considered to be statistically significant.

## Results and discussion

### Celastrol inhibits Aβ_1-42_ induced IKKβ activity

Alzheimer’s disease (AD) is characterized by an extracellular amyloid-β (Aβ) deposition, which activates microglia, triggers neuroinflammation and involves neuronal death and cognitive deficits (Wan et al. 2010). It has been agreed well that the inflammatory response in AD is complex with the role of microglia in the pathogenesis of AD. *In vitro* studies have shown that Aβ directly activates microglia and provokes the secretion of pro-inflammatory cytokines, including IL-1β, IL-6, TNFα (Lucin & Wyss-Coray 2009; Wang et al. 2015).

The burgeoning number of research on inflammation and AD has shown that the pro-inflammatory environment negatively affects the ability of microglial cells to involve in phagocytosis and subsequent retention of Aβ oligomers within the cells (Fiala et al. 2005; Zelcer et al. 2007; Hickman et al. 2008; Yamamoto et al. 2008). A vicious pathophysiological cycle of Aβ and pro-inflammatory cytokines has been observed in AD where inflammatory cytokines act in an autocrine fashion and stimulate β and γ secretases and reduce Aβ clearance by reducing expression of Aβ-binding receptors and Aβ-degrading enzymes and promotes Aβ production (Hickman, et al. 2008); Aβ accumulation further activates microglia-mediated inflammation contributing to disease progression (Wang, et al. 2015). In addition, inflammatory mediators have also reduced the levels of insulin degrading enzyme, an Aβ degrading protease and promote Aβ production (Mushtaq et al. 2015). This might be a secondary mechanism through which inflammation could increase amyloid deposition.

In terms of neuroinflammation, NF-κB activation has been shown to initiate and amplify inflammatory signals by responding to proinflammatory stimuli such as TNF-α or interleukin-1 (IL-1) (Hayden et al. 2006; Glass, et al. 2010). Recent studies have shown that Aβ and APP upregulate NF-κB activity (Granic et al. 2009). Moreover, APP, presenilin and BACE-1 have NF-κB sites in their promoters, and proinflammatory cytokines upregulates their expression in neurons (Sastre et al. 2008).

**Figure 2.**
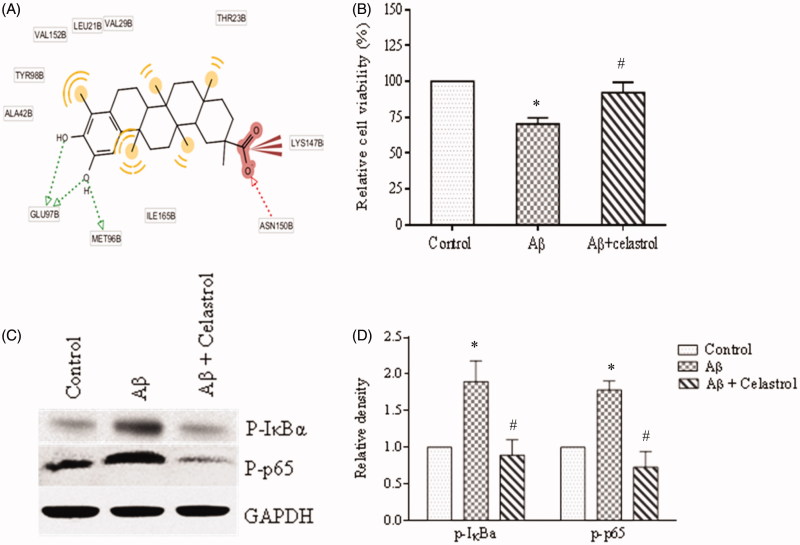
(A) Molecular interaction of IKKβ and celastrol. (B) Effect of celastrol (1 μM) on Aβ 1–42 (10 μM) induced cell death. (C) Effect of celastrol on Aβ 1–42 induced IKKβ activation.

In this study, we observed that Aβ_1-42_ peptide induced the phosphorylation of IκBα and P65 and pretreatment of celastrol at 1 μM concentration inhibits the phosphorylation (Figure 2(C),(D)) in differentiated IMR-32 cells. Celastrol is a well-known inhibitor of NF-κB signalling of natural origin and been used for decades as an anti-inflammatory agent. The decreased expression of pIκBα and pP65 in celastrol pretreated cells affirms the functional representation of inhibited IKKβ activity in these cells. Earlier reports also have shown that celastrol inhibited TNF-α, LPS or PMA induced phosphorylation and degradation of IκBα (Lee et al. 2006). Moreover, Lee et al. (2006) have reported that celastrol inhibited the constitutively active IKKβ without suppressing DNA binding of NF-κB. Collectively, celastrol was observed to prevent IκBα phosphorylation induced by different stimuli via targeting IKKβ, the common step in NF-κB activation pathways.

Since microglia, Aβ, and NF-κB signalling are associated with neuroinflammation in AD, it seems that celastrol, a natural anti-inflammatory product can be an attractive therapy for AD. Given the extensive ongoing research on inflammation and AD, treatment with carefully chosen anti-inflammatory drugs may continue to warrant consideration.

### Molecular docking

Celastrol improved memory, learning and psychomotor activity and suppressed peroxynitrite mediated damage in rat model of AD (Kannaiyan et al. 2011). In our study, celastrol protected differentiated IMR-32 cells against Aβ_1-42_ induced cell death (Figure 2(B)). A recent study by Zhao et al. (2014) also shows that the celastrol has an ability to protect human neuroglioma cells from LPS induced cell death and inhibits Aβ production *in vitro* (Zhao et al. 2014). Although, celastrol has shown to be neuroprotective, it is toxic at increased concentration which is evident from its apoptotic and anti-cancer activity in many cancer cells (Kannaiyan et al. 2011; Raja et al. 2011). Structural modification of celastrol would be promising in an effort to avoid its cytotoxicity without compromising the neuroprotection. We had chosen 36 celastrol analogues and studied plausible molecular interaction with IKKβ by docking.

The analogues chosen for this study were modified either at C-20 carboxylic acid or at A/B rings (C-2, C-3, C-6 positions) of celastrol. Although A/B ring modification offered more active analogues, the intact quinone methide moiety in the A/B ring was reported to be important for both cytotoxicity and neuroprotection (Abbas et al. 2007; Sun, et al. 2010). Considering this, analogues were selected to display diverse modification in all four sites (Figure 1). All the compounds were docked with crystal structure of human IKKβ (PDB 4KIK) (Supplementary Figure 1). The co-crystallized known inhibitor KSA was utilized as control ligand and its binding region (ATP binding pocket located at the hinge loop) with IKKβ was considered as favourable region for inhibition.

The structure of hIKKβ consisted of the N-terminal kinase domain, KD (1–309), the central ubiquitin-like domain, ULD (310–404), and the C-terminal dimerization domain, SDD (408–664). This hinge region (residues 95–100) of KD included three active site residues: Glu97, Tyr98 and Cys99 and these residues were critical for hydrogen-bonding interactions with inhibitors. Celastrol formed two hydrogen bonds with Glu97 and one with Met96, gatekeeper residue which controls the access of inhibitor to binding pocket. An additional hydrogen bond with Asn150 and multiple hydrophobic interactions with Gly loop (residues 20-30) and activation loop (residues 166-194) greatly stabilizes the celastrol IKKβ interaction with the binding score of −10.56 kcal/mol (Figure 2(A)). The analogues were then screened for hydrogen bonding with active site residues and twenty-five of them were selected. The docking poses of all molecules were given in Supplementary Figure 1. Further to confirm IKKβ-celastrol-binding affinities, all docked models were revalidated using X-Score program. The binding affinity score and the X-score results were given in Supplementary Table S1.

Among the selected analogues, seventeen were A/B ring modified while eight were C-20 modified. Thus, analogues were interacted with IKKβ irrespective of intact or modified quinone methide moiety. Twenty-two analogues specifically formed hydrogen bonding with Cys99 residue and compound **28** formed one hydrogen bond with Tyr98. Compound **15** formed two hydrogen bonds (Glu97 and Cys99 each) with highest binding energy of −10.79 kcal/mol. The bindings were also stabilized with multiple nonbonding interaction with the activation loop residues. These compounds were further analyzed for drug like properties and PASS prediction.

### Drug like properties and pass prediction

Despite celastrol has good pharmacological activities, poor solubility and cytotoxicity, it is of major concern to be used as a drug. Hence, in this study, we examined the drug likeness of selected celastrol analogues, implementing computational predictive methods, which inferred to possess anti-inflammatory property and also possible candidates for treating dementia.

Qikprop module from Schrodinger program was utilized to determine the drug-like behaviours of celastrol compounds. The results were evaluated based on their pharmacokinetic properties such as serum protein binding, octanol/water partition coefficient, CNS activity, blood brain barrier level and human oral absorption in gastrointestinal tract. Among these, BBB and CNS, are the most important descriptors in Alzheimer’s drug discovery (Hou et al. 2006; Moroy et al. 2012). The analysis of BBB predicted values showed that celastrol and all analogues were able to cross blood brain barrier but only three compounds showed CNS activity. In addition, no violations of Lipinski rule of 5 were seen in celastrol, compound **2** and **34**. Eight analogues showed one violation but they can be considered for the drug development as they are derived from natural products (Ganesan 2008). The detailed ADMET result of celastrol molecules was shown in Supplementary Tables S2 and S3.

Recently, several computational tools were used to identify potential targets from natural product molecules (Lagunin et al. 2010). In this study, we identified anti-inflammatory, neurotrophic, cytoprotectant and dementia treatment as biological functions for celastrol and its analogues by implementing PASS program. This program predicts the biological activity spectrum based on the chemical structure formula. The celastrol anti-inflammatory potency was retained in all the compounds. Similarly, most of the analogues were observed to be nootropic and cytoprotectant but only for celastrol, compound **2** and **34** showed high Pa value for dementia treatment. The results were shown in Supplementary Table S4. Collectively, compound **15** has the highest binding affinity, CNS activity and more drug likeness than parent celastrol. Although this study is an initial screening using *in silico* approach, further studies are needed to confirm the potency of compound **15** and other celastrol analogues for IKKβ inhibition and neuroprotection.

## Conclusions

Overall, IKKβ is an attractive anti-inflammatory target in neurodegenerative diseases. Celastrol, a known NF-κB inhibitor modulates NF-κB signalling through inhibition of IKKβ. The present study revealed the binding pattern of celastrol and its analogues within the active site of IKKβ. Molecular-docking analysis displayed 25 celastrol molecules located well within the ATP-binding site. Each compound formed at least one hydrogen-bond interaction with active site residues Glu97, Tyr98 and Cys99. Non-bonded interactions were also seen with activation loop residues stabilizing the ligand and IKKβ interaction. Analysis of drug-like properties demonstrated few compounds were capable of crossing BBB, non-toxic and obeyed softened Lipinski rule of 5. The biological property prediction revealed that celastrol anti-neuroinflammatory and neuroprotective properties have been retained in its analogues. Further experimental studies are needed to validate the IKK inhibition and neuroprotection of selected celastrol analogues.

## Supplementary Material

Shila_Samuel_et_al_supplemental_content.zip
