# Supplementary material for: Inhibition of IKKβ by celastrol and its analogues – an in silico and in vitro approach
Source: Pharm Biol. 2016 Dec 8;55(1):368–73. doi: 10.1080/13880209.2016.1241809 (PMC6130723; doi:10.1080/13880209.2016.1241809)
Supplement: Shila_Samuel_et_al_supplemental_content.zip [file IPHB_A_1241809_SM0405.zip › Shila Samuel et al supplemental content.pdf]

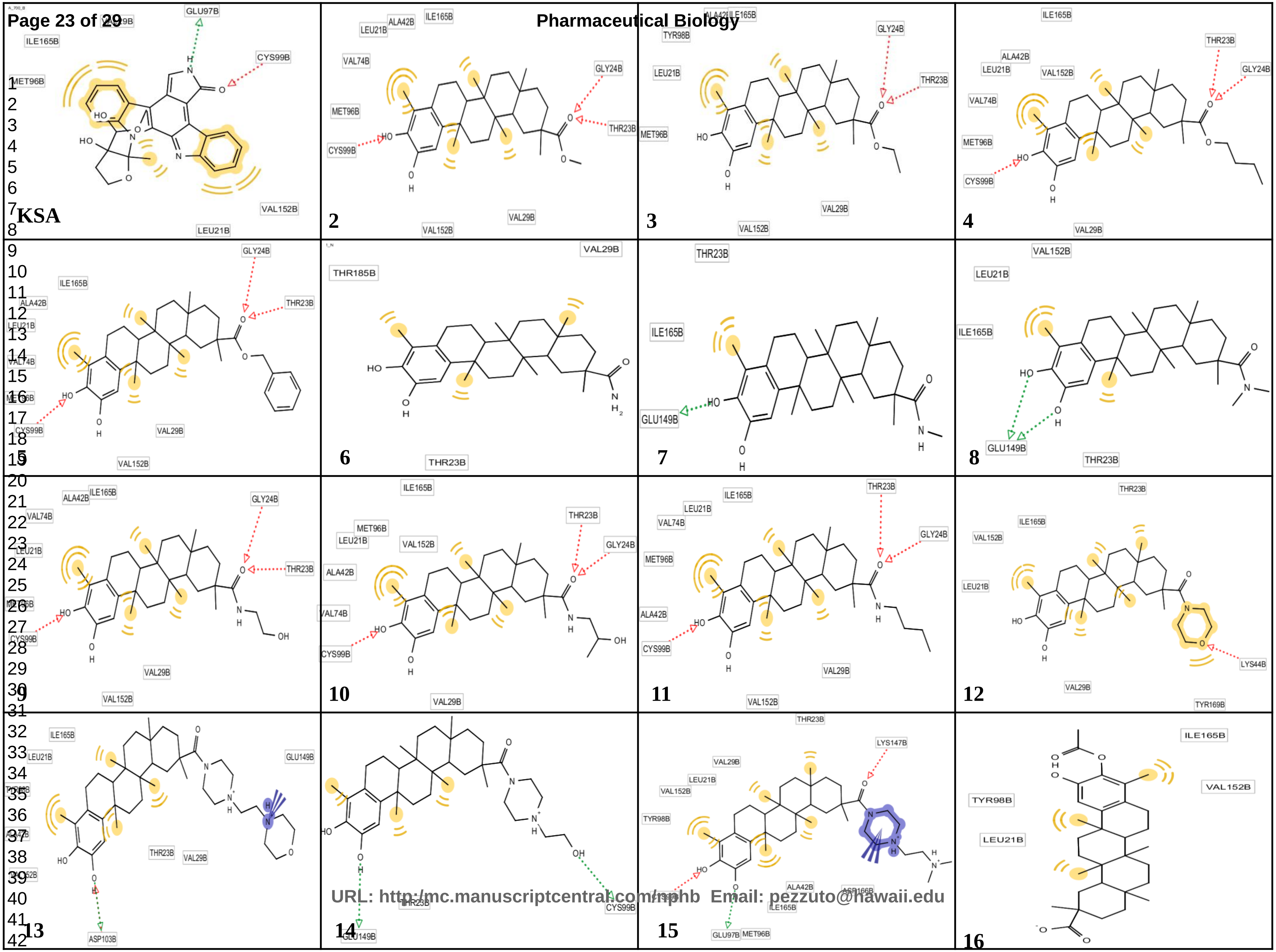
KSA
GLU97B
CYS99B
ILE165B
MET96B
VAL152B
LEU21B
2
ALA42B
ILE165B
LEU21B
VAL74B
MET96B
CYS99B
GLY24B
THR23B
VAL29B
VAL152B
3
TYR98B
LEU21B
MET96B
GLY24B
THR23B
VAL29B
VAL152B
4
ILE165B
THR23B
ALA42B
LEU21B
VAL152B
GLY24B
VAL74B
MET96B
CYS99B
VAL29B
5
GLY24B
ILE165B
THR23B
ALA42B
LEU21B
VAL74B
CYS99B
VAL29B
VAL152B
6
THR185B
VAL29B
THR23B
7
THR23B
ILE165B
GLU149B
8
VAL152B
LEU21B
ILE165B
GLU149B
THR23B
9
ALA42B
ILE165B
GLY24B
VAL74B
LEU21B
THR23B
CYS99B
VAL29B
VAL152B
10
ILE165B
MET96B
LEU21B
VAL152B
THR23B
GLY24B
ALA42B
VAL74B
CYS99B
VAL29B
11
ILE165B
LEU21B
THR23B
VAL74B
GLY24B
MET96B
ALA42B
CYS99B
VAL29B
VAL152B
12
THR23B
ILE165B
VAL152B
LEU21B
LYS44B
VAL29B
TYR169B
13
ILE165B
LEU21B
GLU149B
THR23B
VAL29B
ASP103B
14
GLU149B
CYS99B
15
THR23B
LYS147B
VAL29B
LEU21B
VAL152B
TYR98B
ALA42B
ILE165B
GLU97B
MET96B
16
ILE165B
VAL152B
TYR98B
LEU21B

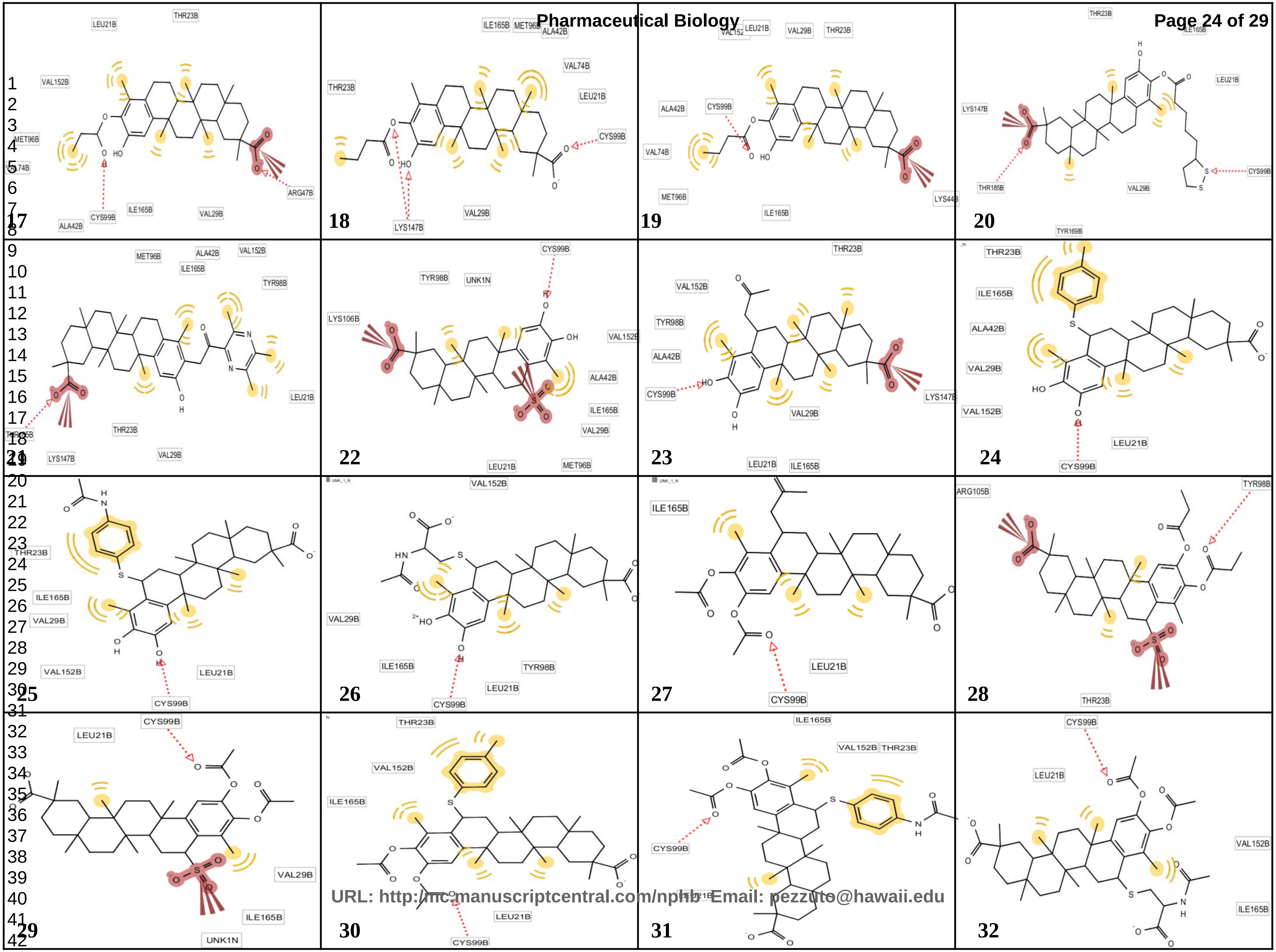
17
LEU21B
THR23B
VAL152B
MET96B
VAL74B
ARG47B
ILE165B
VAL29B
CYS99B
ALA42B
18
ILE165B
MET96B
ALA42B
VAL74B
THR23B
LEU21B
CYS99B
VAL29B
LYS147B
19
LEU21B
VAL29B
THR23B
ALA42B
CYS99B
VAL74B
MET96B
ILE165B
20
THR23B
LEU21B
LYS147B
THR185B
VAL29B
CYS99B
TYR169B
21
MET96B
ALA42B
VAL152B
ILE165B
TYR98B
LEU21B
THR23B
LYS147B
VAL29B
22
CYS99B
TYR98B
UNK1N
LYS106B
ALA42B
ILE165B
VAL29B
LEU21B
MET96B
23
THR23B
VAL152B
TYR98B
ALA42B
CYS99B
VAL29B
LEU21B
ILE165B
24
THR23B
ILE165B
ALA42B
VAL29B
VAL152B
LEU21B
CYS99B
25
THR23B
ILE165B
VAL29B
VAL152B
LEU21B
CYS99B
26
VAL152B
VAL29B
ILE165B
TYR98B
LEU21B
CYS99B
27
ILE165B
LEU21B
CYS99B
28
ARG105B
TYR98B
THR23B
29
CYS99B
LEU21B
VAL29B
ILE165B
UNK1N
30
THR23B
VAL152B
ILE165B
LEU21B
CYS99B
31
ILE165B
VAL152B
THR23B
CYS99B
32
CYS99B
LEU21B
VAL152B
ILE165B

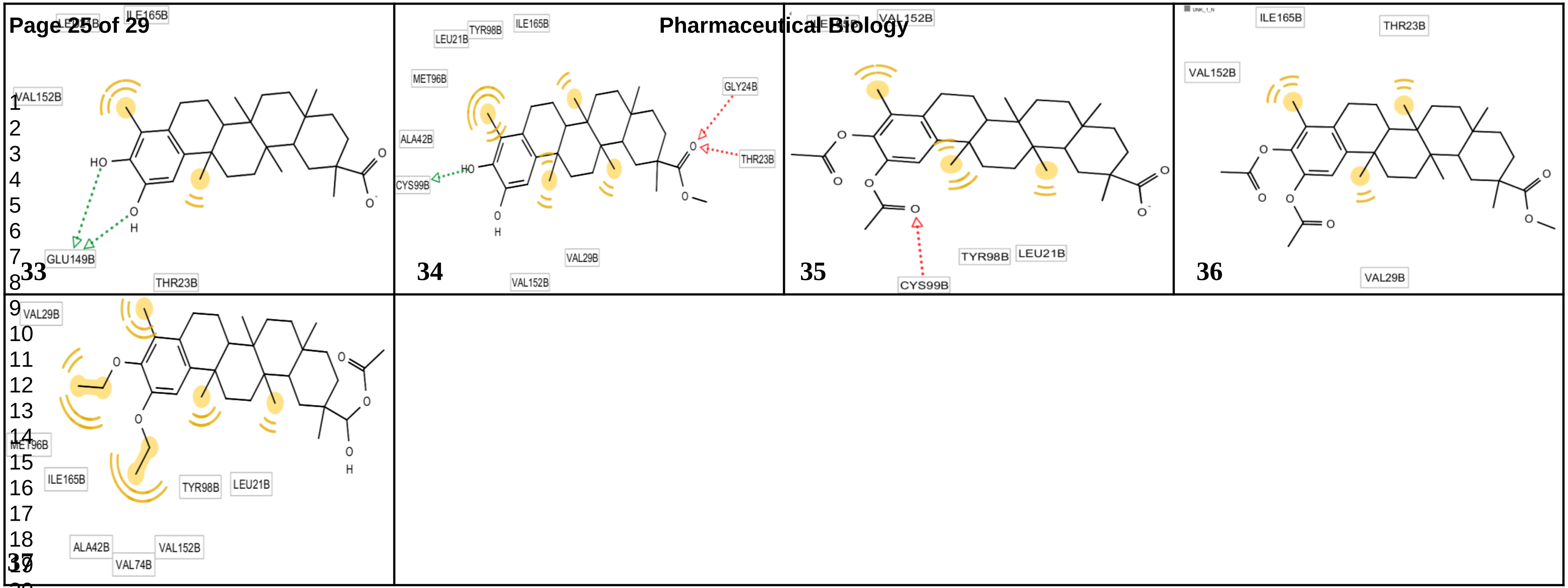

Supplementary Figure 1. Graphical illustration of docked complexes of IKKβ with KSA and celastrol compounds. The pharmacophore features present in each docked complexes were highlighted in yellow colors and active site residues, hydrogen bonds interactions were colored by LigandScout program by using default parameters.

Table S1: Molecular interaction results and binding affinity of IKKβ with celastrol analogous. The docking scores were re-evaluated using X-score program.

Molecule	Predicted binding affinity (kcal/mol)[a]	Hydrophobic pair score (pK_d)[a]	Hydrophobic match score (pK_d)[a]	Hydrophobic surface score (pK_d)[a]	Predicted mean binding affinity (pK_d)[a]
KSA	-10.76	7.93	8.4	7.33	7.88
Celastrol	-10.56	7.72	8.02	7.48	7.74
2	-10.30	7.49	7.85	7.3	7.55
3	-10.60	7.54	8.39	7.38	7.77
4	-10.14	7.24	7.97	7.1	7.44
5	-10.37	7.39	8.03	7.38	7.6
6	-8.56	5.8	6.67	6.35	6.27
7	-8.37	5.62	6.91	5.87	6.13
8	-8.51	5.81	6.91	5.99	6.24
9	-10.14	7.39	7.71	7.2	7.43
10	-10.22	7.33	7.93	7.22	7.49
11	-10.38	7.43	8.05	7.34	7.61
12	-9.87	7.02	7.52	7.17	7.23
13	-10.47	7.58	7.96	7.5	7.68
14	-8.06	5.28	7.03	5.43	5.91
15	-10.79	7.95	8.14	7.65	7.91
16	-9.09	6.36	7.21	6.42	6.66
17	-10.08	7.07	7.73	7.37	7.39
18	10.26	7.51	7.99	7.05	7.52
19	-10.11	7.18	7.59	7.47	7.41
20	-10.43	7.22	8.11	7.60	7.64
21	-10.36	7.36	7.94	8.12	7.81
22	-9.99	7.17	8.07	6.74	7.33
23	-10.38	7.47	8.06	7.3	7.61
24	-10.60	7.54	8.39	7.38	7.77
25	-10.76	7.62	8.64	7.42	7.89
26	-10.14	7.34	7.96	7	7.43
27	-10.17	7.04	8.1	7.21	7.45
28	-8.41	5.63	6.95	5.91	6.16
29	-9.83	6.82	8.03	6.76	7.2
30	-10.71	7.45	8.49	7.62	7.85
31	-10.76	7.43	8.67	7.57	7.89
32	-10.19	7.14	8.18	7.1	7.47
33	-8.55	5.79	7.07	5.95	6.27
34	-10.20	7.25	8.03	7.15	7.48
35	-9.96	6.91	8.09	6.9	7.3
36	-9.74	6.66	7.57	7.19	7.14
37	-9.72	6.75	7.94	6.67	7.12

For Peer Review Only

Table S2: List of pharmacophore features involved in each docking confirmation were analyzed by Ligandsout v3.1 program.

S.No	Molecule	Pharmacophore features					
		**Total number of features**	**H-bond donor**	**H-bond Acceptor**	**Hydrophobic**	**Neg ionizable**	**Pos ionizable**
1	Celastrol	10	3	1	5	1	-
2	2	7	-	3	4	-	-
3	4	7	-	3	4	-	-
4	5	7	-	3	4	-	-
5	9	7	-	3	4	-	-
6	10	7	-	3	4	-	-
7	11	7	-	3	4	-	-
8	14	5	2	-	3	-	-
9	15	8	1	2	4	-	1
10	17	8	-	2	5	1	-
11	18	8	-	3	5	-	-
12	19	7	-	1	5	1	-
13	20	6	-	2	3	1	-
14	22	6	-	1	3	2	-
15	23	7	-	1	5	1	-
16	24	6	-	1	5	-	-
17	25	5	-	1	4	-	-
18	26	4	-	1	3	-	-
19	27	4	-	1	3	-	-
20	28	5	-	1	2	2	-
21	29	4	-	1	2	1	-
22	30	6	-	1	5	-	-
23	31	4	-	1	3	-	-
24	32	4	-	1	3	-	-
25	34	7	1	2	4	-	-
26	35	4	-	1	3	-	-

For Peer Review Only

Table S3: ADMET results of various celastrol molecules with pharmacokinetic properties.

S.No	Molecule	MW	QPlogBB	QP (%)	QPlogKhsa	CNS	QPlogPo/w	Lipinski Rule of 5 violations
1	Celastrol	450.6	-1.2	85	1.0	-2	4.8	0
2	2	464.6	-0.8	94	1.4	-1	5.3	1
3	4	506.7	-1.1	88	1.8	-2	6.5	2
4	5	540.7	-1.1	92	2.0	-2	7.1	2
5	9	493.6	-1.5	85	0.7	-2	3.7	0
6	10	507.7	-1.4	78	0.8	-2	4.1	1
7	11	505.7	-1.0	80	1.3	-2	5.5	2
8	14	562.7	-1.1	62	0.8	-2	3.5	1
9	15	589.8	-0.3	63	1.0	1	4.1	1
10	17	506.6	-1.1	67	1.1	-2	5.5	2
11	18	520.6	-1.3	68	1.3	-2	5.9	2
12	19	520.6	-1.2	69	1.3	-2	5.9	2
13	20	638.9	-1.4	81	1.8	-2	7.9	2
14	22	554.6	-2.1	43	0.4	-2	4.0	1
15	23	508.6	-1.7	56	1.1	-2	5.2	2
16	24	574.8	-1.5	75	1.8	-2	7.3	2
17	25	617.8	-2.3	59	1.5	-2	6.4	2
18	26	613.8	-2.9	39	0.6	-2	4.8	1
19	27	592.7	-1.6	65	1.2	-2	5.8	1
20	28	666.7	-2.4	45	0.7	-2	5.6	2
21	29	638.7	-2.0	51	0.4	-2	4.6	1
22	30	658.8	-1.2	84	2.0	-2	8.0	2
23	31	701.9	-1.3	76	1.5	-2	6.9	2
24	32	697.8	-1.8	51	0.5	-2	5.6	2
25	34	466.6	-0.8	100	1.6	-1	5.8	0
26	35	536.6	-1.2	68	1.3	-2	5.8	2

MW: Molecular weight; QPlogBB: Log of Blood Brain Passage Value (acceptable range: -3.0 / 1.2); QP (%): Percentage of human oral absorption in GI (acceptable range: <25% is poor and >80% is high); QPlogKhsa: Serum protein binding (acceptable range: -1.5 / 1.5); CNS: Predicted central nervous system activity on a –2 (inactive) to +2 (active) scale; QPlogPo/w: Octanol/water partition coefficient (acceptable range -0.2 to 6.5).

For Peer Review Only

Table S4: Pass predicted activity scores of selected celastrol analogues.

S. No	Molecules	Anti - inflammatory		Nootropic		Cytoprotectant		Dementia treatment	
		Pa	Pi	Pa	Pi	Pa	Pi	Pa	Pi
1	Celastrol	0,845	0,005	0,755	0,026	0,606	0,028	0,350	0,088
2	2	0,819	0,005	0,605	0,075	0,563	0,044	0,380	0,064
3	4	0,805	0,006	0,636	0,061	0,578	0,038	0,296	0,148
4	5	0,803	0,006	0,540	0,107	0,563	0,044	0,301	0,140
5	9	0,658	0,021	0,547	0,102	0,474	0,070	NA	NA
6	10	0,578	0,037	0,683	0,046	0,424	0,087	0,250	0,222
7	11	0,632	0,026	0,622	0,067	0,585	0,036	NA	NA
8	14	0,656	0,022	0,572	0,089	0,322	0,160	0,244	0,235
9	15	0,562	0,040	0,396	0,220	0,259	0,248	NA	NA
10	17	0,862	0,005	0,745	0,028	0,579	0,038	0,246	0,230
11	18	0,850	0,005	0,747	0,028	0,627	0,021	NA	NA
12	19	0,840	0,005	0,606	0,074	0,476	0,070	0,276	0,178
13	20	0,861	0,005	NA	NA	0,364	0,120	NA	NA
14	22	0,828	0,005	NA	NA	0,391	0,103	NA	NA
15	23	0,717	0,014	0,465	0,160	0,590	0,034	NA	NA
16	24	0,882	0,005	0,465	0,161	0,381	0,108	NA	NA
17	25	0,841	0,005	0,410	0,206	0,264	0,241	NA	NA
18	26	0,826	0,005	0,610	0,072	0,490	0,066	NA	NA
19	27	0,759	0,009	0,433	0,185	0,633	0,019	NA	NA
20	28	0,845	0,005	NA	NA	0,353	0,129	NA	NA
21	29	0,843	0,005	NA	NA	0,495	0,064	NA	NA
22	30	0,890	0,004	0,433	0,184	0,480	0,068	NA	NA
23	31	0,854	0,005	0,383	0,233	0,312	0,172	NA	NA
24	32	0,841	0,005	0,588	0,082	0,562	0,044	NA	NA
25	34	0,800	0,007	0,680	0,046	0,538	0,052	0,355	0,084
26	35	0,846	0,005	0,789	0,019	0,634	0,019	0,253	0,217

For Peer Review Only
